# Disposition of a single oral dose of a cannabidiol medication in healthy cats

**DOI:** 10.3389/fvets.2023.1181517

**Published:** 2023-05-26

**Authors:** Tom Jukier, Crisanta Cruz-Espindola, Doug Martin, Dawn M. Boothe

**Affiliations:** ^1^Department of Clinical Sciences, College of Veterinary Medicine, Auburn University, Auburn, AL, United States; ^2^Clinical Pharmacology Laboratory, Department of Anatomy and Physiology, College of Veterinary Medicine, Auburn University, Auburn, AL, United States; ^3^Scott Ritchey Research Center, Department of Anatomy and Physiology, College of Veterinary Medicine, Auburn University, Auburn, AL, United States

**Keywords:** cannabidiol, CBD, cat, epilepsy, seizures

## Abstract

The historical use of the marijuana plant for medicinal purposes is long. One of the historical uses has been for the treatment of epilepsy. Recently, the Food and Drug Administration has approved a highly purified cannabidiol medication for the add on therapy in people with certain forms of epilepsy. With the increase interest of the use of cannabidiol in the veterinary community, the aim of this study was to describe the disposition of a single dose of a cannabidiol medication in healthy cats in both the fed and fasted state. Pharmacokinetic analysis reveals that relative bioavailability of cannabidiol shows a near eleven-fold increase when administered in the fed state compared to the fasted state. Additionally, concentrations achieved at a dose of 5 mg/kg, may be sufficient to explore the therapeutic potential in cats with epilepsy.

## Introduction

Marijuana (*Cannabis* spp.) has a long history of medical use (1, 2). However, understanding its efficacy did not begin until its two major phytocannabinoids, cannabidiol (CBD) and Δ9-tetrahydrocannabinol (THC), were discovered in 1940 (3) and 1964 (4), respectively. Of these two, CBD has recently gained attention for its possible therapeutic effects, while avoiding some of the undesirable THC-related psychotropic effects. Among the therapeutic indications of CBD is epilepsy. Evidence of efficacy in rodent models of epilepsy (5–9) led to research in humans. Subsequently, CBD has demonstrated efficacy in the treatment of Lennox-Gastaut (10, 11) and Dravet (12) epileptic syndromes. These studies culminated in the 2018 Food and Drug Administration (FDA) approval of a highly purified form of CBD (EPIDIOLEX^®^ Jazz Pharmaceuticals Inc., Palo Alto, CA, USA). Subsequently, EPIDIOLEX^®^ has been additionally approved for the treatment of tuberous sclerosis complex epileptic syndrome (13).

CBD appears to be characterized by complex pharmacokinetics that may prove challenging when attempting to achieve therapeutic concentrations. Notably, first-pass metabolism has been demonstrated in multiple species, including humans (14, 15). Among the approaches which might maximize oral absorption is administering CBD with food. In humans, the area under the curve (AUC) and maximum serum concentration (C_max_) of CBD are increased by 5- and 3-fold, respectively, when administered with food (16).

While the body of CBD literature in veterinary medicine is increasing, information about the disposition of CBD is limited to cats. The high tolerability of a CBD supplement in cats was recently demonstrated with doses of up to 30 mg/kg, with only mild adverse effects being noted (17). There is a need for CBD pharmacokinetic studies in cats. Among the challenges of determining a CBD dosing regimen is the difficulty in achieving effective concentrations following oral administration. Paramount to determining the efficacy of any medication is maximizing the likelihood of the dosing regimen in achieving therapeutic concentrations through pharmacokinetic studies in the species of interest.

Another challenge impacting the safe and effective use of CBD for the treatment of epilepsy in cats is the source of CBD that might be used for treatment. Currently, only reports of CBD supplements are being used. Because supplements marketed to animals undergo no federally mandated regulatory assessment for quality, the accuracy of the labels and the product contents is not assured (18). An advantage of using an FDA-approved CBD product is that such concerns can be avoided. Furthermore, the concentration of the product (100 mg/ml) is sufficiently high that dosing volumes are tenable. Finally, because the drug is FDA-approved, federal law (the Animal Medicinal Drug Use Clarification Act) provides a legal pathway for veterinarians in the United States of America to use CBD, albeit with an extra label for their patients. The primary aim of this study was to determine the disposition of single oral dose Epidiolex^®^.

## Materials and methods

All protocols were approved by the Institutional Animal Care and Use Committee of Auburn University. A total of nine healthy, adult cats (4 male and 5 female) ranging in age from 1 to 7 years were obtained from a breeding colony. Cats were studied using a randomized cross-over design in which cats received a single 5 mg/kg of CBD either in a fasted state or 30 min after feeding a commercial balanced diet. All cats received a physical and neurological examination before the study. Male cats were fed Hill's dry C/D (Hill's Pet Nutrition, Topeka Kansa, USA) and Friskies (PURINA, Neenah, WI, USA), while female cats were fed Hill's adult food (Hill's Pet Nutrition, Topeka Kansa, USA) and Friskies (PURINA, Neenah, WI, USA). Serum biochemistry was performed in each cat before the start of the first phase of the study. Results and reference intervals are available in Supplementary material.

The day before the study, jugular catheters (MILA International INC., Florence, Kentucky, USA) were placed under anesthesia using 4 mcg/kg of dexmedetomidine (Dexdomitor^®^ 0.5 mg/mL, Orion Corporation, Espoo, Finland) and 10 mg/kg of ketamine (Ketamine hydrochloride injection 100 mg/mL, Covetrus, North America, Dublin, OH) intramuscularly followed by intubation and isoflurane. Cats were fasted for 10 h before the start of the study if they were placed into the fed group, and an additional 4 h if they were in the fasted group. If allocated to the fed group, cats were fed their regular diet approximately 30 min before drug administration. CBD was administered *via* the 1-mL syringes and directly injected into the cat's oral cavity. Approximately 3 mL of blood was drawn at 0, 0.25, 0.5, 1, 2, 4, 6, 8, 10, 12, 24, and 48 h after dosing. Blood was transferred into a lithium heparin tube (S-Monovette^®^, SARSTEDT AG & Co. KG, Numbrecht, Germany) and immediately placed on ice. Samples were centrifuged within 2 h of collection at 2,500 *g* for 10 min, and plasma was harvested and stored in a −80°C freezer until analysis. The cross-over phase of the study was performed 30 days later, with each cat receiving its alternate assigned treatment (fed vs. fasted).

Feline plasma was analyzed for cannabidiol (CBD) and Δ9-Tetrahydrocannabinol (Δ9-THC) using a liquid chromatography–tandem mass spectrometry triple quadrupole (LC-MS/MS) (19–25). The system consisted of a UPLC Acquity system coupled with a Triple Quad Mass Spectrometer detector (Waters™, Milford, MA, USA) (19–21) equipped with an electrospray ionization (ESI) source (19, 20, 23, 25). Detection and quantification were conducted using multiple reaction monitoring (MRM). The transition of the precursor ion (m/z) and the product ion (m/z) for CBD, Δ9-THC, and Δ9-THC-D3 (internal standard) were 315.3/193.2, 315.2/193.1, and 318.2/196.1), respectively. Data acquisitions were done using MassLynx 4.1 software (Waters™, Milford, MA, USA) (19). The mass spectrometer was operated in positive ionization mode (19, 20, 22). Separation of the cannabinoids was achieved with a Waters Acquity UPLC BEH C18, 1.7 μm, 2.1 X 50 mm column (19, 21–23, 25) (Waters™, Milford, MA, USA), at 45°C (19, 21). The mobile phase consisted of 0.1% formic acid and acetonitrile (VWR^®^, Radnor, PA, USA) (19, 21) using a gradient (run 1.5 min at 70% of acetonitrile, then change to 90% at 2.8 min, and then back to the initial conditions at 3 min) with the flow rate set to 0.5 mL/min (19, 21–23).

Cannabinoid standards for feline plasma analysis were made by adding known amounts of CBD and Δ9-THC (Cerilliant, a Sigma-Aldrich^®^ company, Round Rock, TX, USA) (19, 21, 22, 25) stock solution in methanol to feline plasma (Animal Blood Resources International, Stockbridge, MI, USA). The standard curve was generated for both CBD and Δ9-THC ranging from 0.98 to 1,000 ng/mL by fortifying feline plasma with known amounts of CBD, Δ9-THC as a reference standard, and Δ9-THC-D3 (Cerilliant, a Sigma-Aldrich^®^ company, Round Rock, TX, USA) (19, 21, 22, 24, 25) as an internal standard. A standard curve was accepted if the coefficient of determination (r^2^) was at least 0.99 and the predicted concentrations were within 20% of the actual concentrations for the small concentrations and 10% for the high concentrations. The cannabinoids were extracted from feline plasma with an Oasis Prime HLB, 1 cc, 30 mg solid phase extraction (SPE) cartridges (Waters Corporation™, Milford, MA, USA) (19, 21–23). In brief, previously frozen plasma samples were thawed and vortexed. Plasma feline samples were pre-treated for solid phase extraction by combining 250 μL of plasma with 250 μL of water, and the addition of 25 μL of internal standard (1,000 ng/mL) (19, 21–24). The tube was vortexed, and then, 500 μL of 0.1% formic acid in acetonitrile (VWR^®^, Radnor, PA, USA) was added to each tube (19–21, 24). The tube was vortex mixed for 30 s and centrifuged at 3,000 rpm for 12 min at 5°C. The supernatant (800 μL) was loaded into the SPE cartridge and allowed to elute with the vacuum. The cartridges were rinsed with 500 μL of water and then with 500 μL of 5:95 (v/v) methanol:water. The cartridges were dry under vacuum to remove the residual solvent. Cannabinoids were eluted with 500 μL of 90:10 (v/v) acetonitrile:methanol and then evaporated to dryness under a stream of nitrogen for 12 min at 40°C (19–22). The residue was reconstituted with 50 μL of methanol and vortex mixed for 20 s, and then, the solution was centrifuged at 14,000 rpm for 10 min at room temperature. A clear supernatant of 45 μL was transferred to a sampling vial for the LC-MS. The injection volume was 2 μL. The retention time for CBD and Δ9-THC was 0.99 min and 1.78 min, respectively.

The LOD for both cannabinoids was 0.98 ng/mL, and the LLOQ was 1.95 ng/mL. The linear correlation coefficient for CBD and Δ9-THC was 0.999. The precision for CBD in feline plasma at 3.91, 15.63, 125, and 500 ng/mL was 14.64, 10, 5.8, and 4.37%, respectively. The precision for Δ9-THC in feline plasma 3.91, 15.63, 125, and 500 ng/mL was 7.98, 5.63, 3.30, and 3.25%, respectively. The accuracy (% recovery) for CBD and Δ9-THC was 101.43 ± 2.9 and 102.06 ± 2.8, respectively.

### Pharmacokinetic analysis

Plasma CBD concentrations vs. time curves were analyzed by non-compartmental analysis using pharmacokinetic software (Phoenix WinNonlin^®^, Pharsight Corporation, Mountain View, California, USA). Non-compartmental analysis was performed using the linear-log up-down trapezoidal option for the determination of the area under the concentration (AUC) vs. time (Phoenix WinNonlin^®^). From this, the following were determined: mean residence time (MRT), disappearance rate constant (k_d_), terminal half-life (t_1/2_, determined from the relationship t_1/2_ = 0.693/k_el_), area under the curve to infinity (AUC_∞_), percent of the AUC that was extrapolated from the terminal component of the curve (AUC ext), and, in the absence of IV administration, the ratios of apparent volume of distribution to bioavailability (Vz/F) and clearance to bioavailability (Cl/F). Maximum plasma drug concentration (C_max_) and time to maximum concentration (T_max_). Data were reported both as a mean and standard deviation (SD) and as median and range (minimum and maximum values). Relative bioavailability was calculated based on an individual basis using (AUC_FED_*DOSE_FASTED_)/(AUC_FASTED_*DOSE_FED_) × 100.

The Kolmogorov–Smirnov test was performed to assess the normality of the data. All samples were normally distributed except for the C_max_ and AUC for the fasted group. All normally distributed data were reported as mean +/– standard deviation, while non-normal data were reported as median and IQR (C_max_ and AUC for fasted cats). The medians for C_max_ and AUC were compared using a Mann–Whitney *U*-test, while T_max_, disappearance half-life, and MRT were compared using student's *t*-test. Statistical significance was set at an α <0.05. Statistical analysis was performed using Minitab 17 Statistical Software (2010) [Computer software] (State College, PA: Minitab, Inc.).

## Results

Due to catheter displacement, data were available only for one route and one cat and therefore were excluded from the pharmacokinetic analysis.

**Figure 1:**
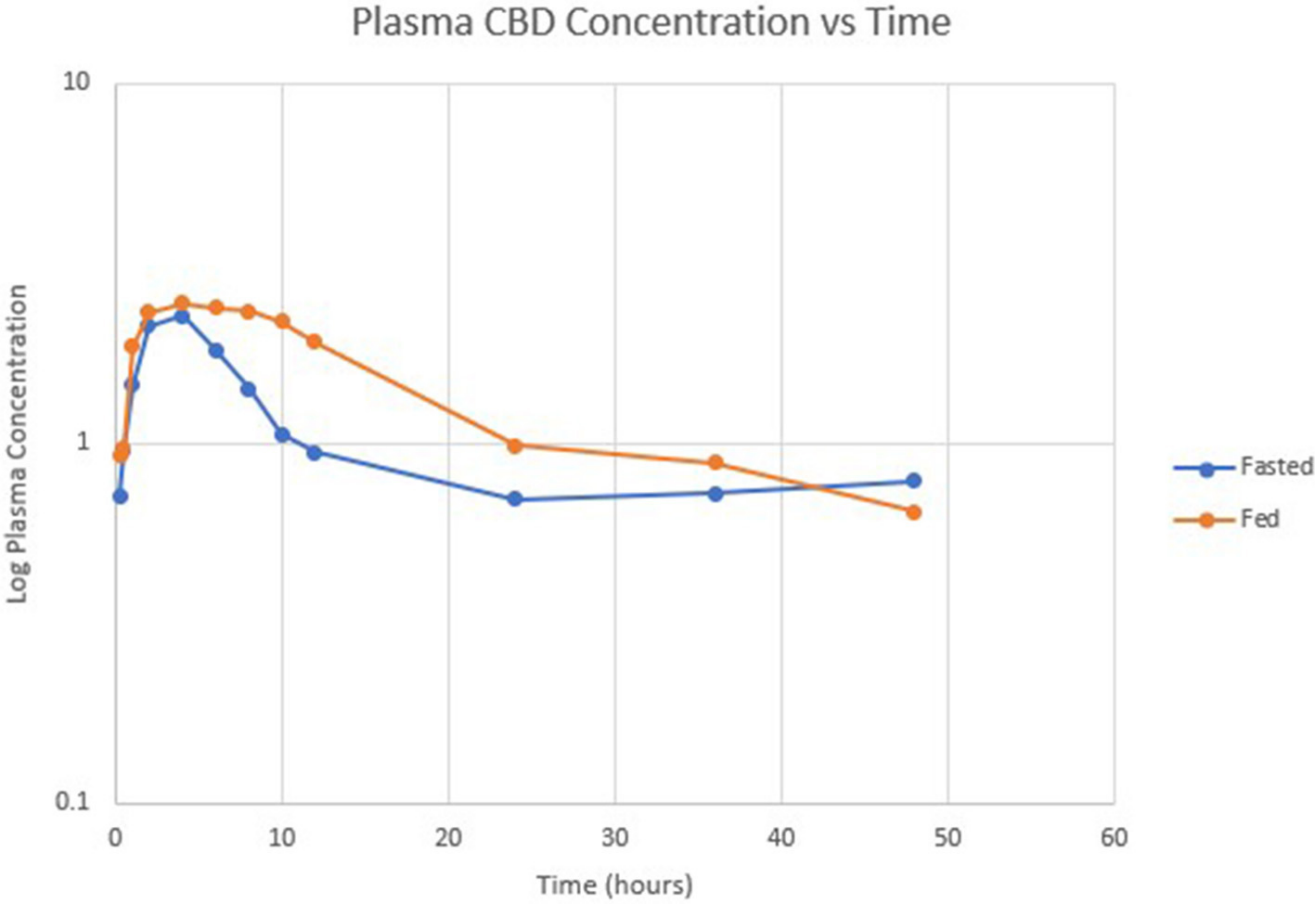
Semi-logarithmic scale showing mean CBD concentration vs. time in fasted (blue) and fed (orange) states.

**Figure 2:**
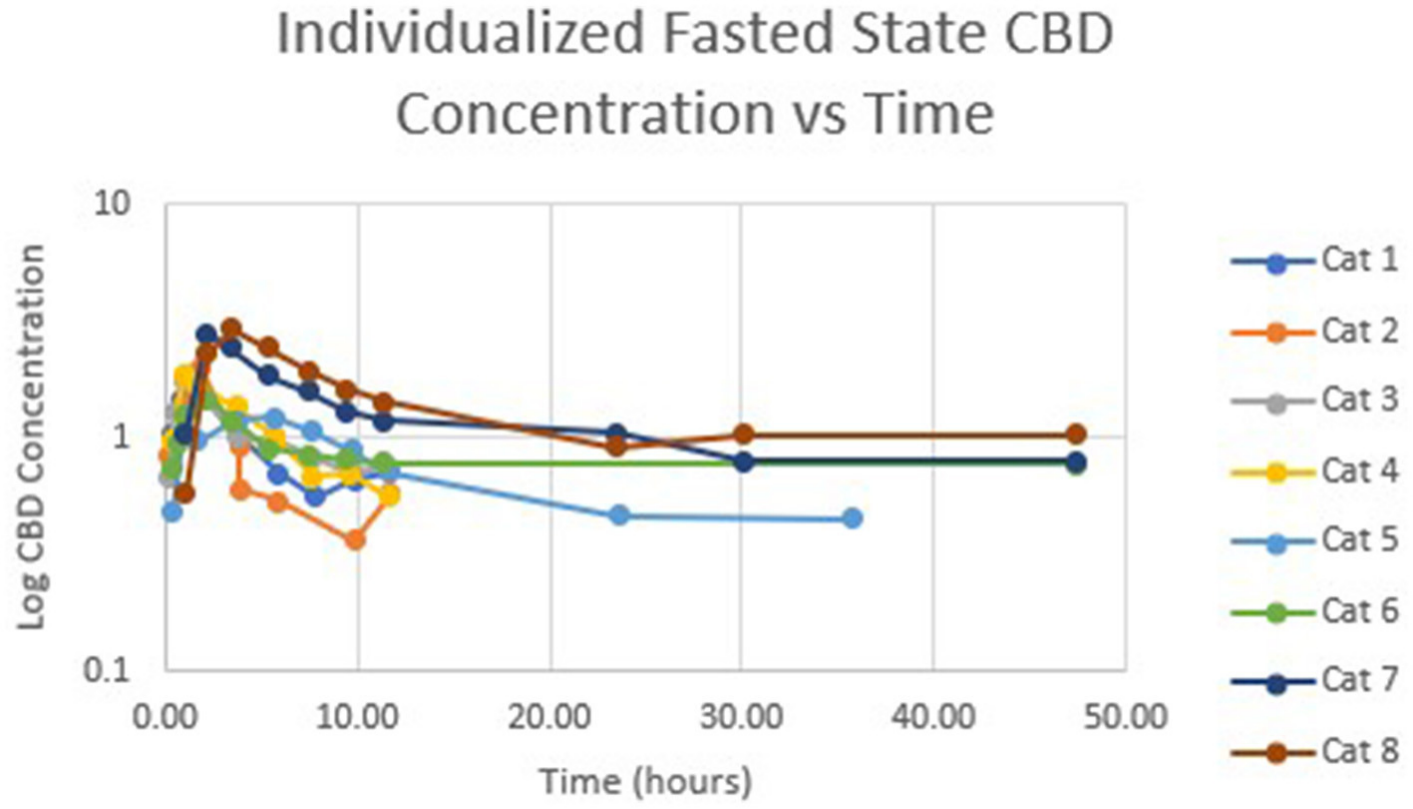
Semi-logarithmic representation of CBD concentration vs. time for indicidual cats administered a 5 mg/kg dose of CBD in a fasted state.

**Figure 3:**
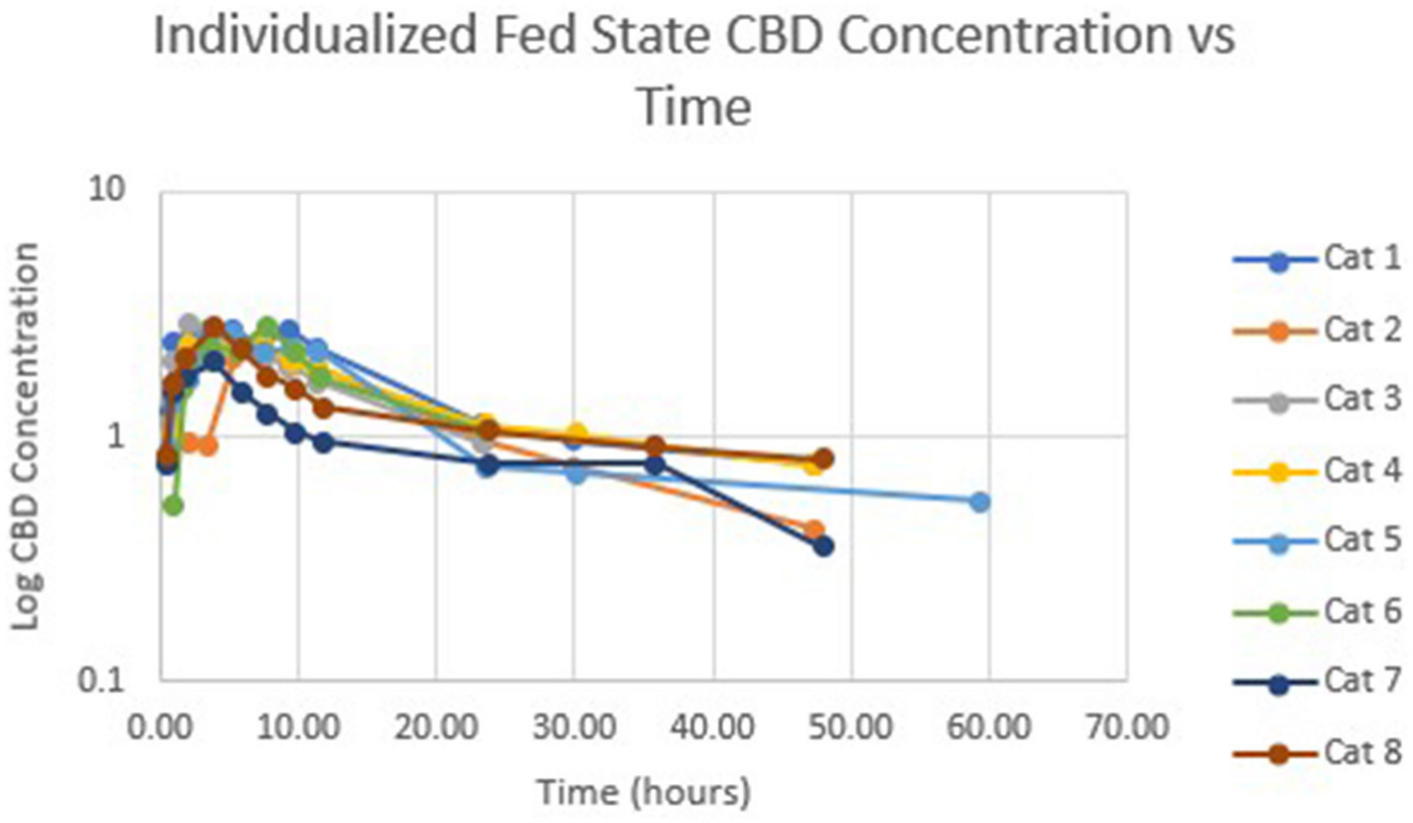
Semi-logarithmic representation of CBD concentration vs. time for indicidual cats administered a 5 mg/kg dose of CBD in a fed state.

Table 1 summarizes pharmacokinetic data. In the fed state, the values for Cmax and AUC appeared to be higher than when administered in the fed state, while Tmax appeared to be longer in the fed state than in the fasted state. The means for terminal half-life and MRT did not appear different. Statistically significant differences between the fasted and fed groups were restricted to AUC (*p* = 0.01) and T_max_ (*p* = 0.036). Figure 1 shows mean concentrations of CBD in a fasted vs. fed state, while Figures 2, 3 display log CBD concentrations vs. time for individual cats in the fasted and fed states, respectively. Absorption was demonstrated to have a near eleven-fold (mean = 10.8) increase in relative oral bioavailability of CBD based on the means of individual AUC in the fed and fasted states. All cats in the fed group achieved C_max_ plasma concentration of >100 ng/mL, with 7/8 achieving plasma concentrations of >200 ng/mL.

**Table 1:** Summary statistics of pharmacokinetic parameters for the fasted vs. fed group.

**Cat group**	**Cmax^1^ (ng/mL) Mean ±SD**	**Tmax^2^ (Hours) Mean ±SD^†^**	**Terminal half-life harmonic Mean ±Psuedo-SD**	AUC^3^ (h^*^ng/mL) Mean ±SD^†^	**MRT^4^ (Hours) Mean ±SD**	**VzF^5^ (L/Kg) Mean ±SD**	**CLssF^6^ (L/h/kg) Mean ±SD**
Fasted	269 ± 334^*^	2.6 ± 1.6	4.1 ± 4.4	921 ± 1,003^**^	12.1 ± 7.5	668.3 ± 1,130	16 ± 10.1
Fed	465.3 ± 220	4.7 ± 2.1	5.9 ± 2	2,650 ± 1,188	10.2 ± 2.4	76.5 ± 66.8	2.7 ± 2.3
Relative bioavailability	10.8						

1 Maximal concentration.

2 Time to maximal concentration.

3 Area under the time concentration curve.

4 Mean residence time.

5 Apparent volume of distribution not corrected for bioavailability.

6 Clearance not corrected for bioavailability.

† Indicates a statistically significant difference between the groups.

* Nonparametric data, median (IQR) = 65 (31–589). For comparison C_max_ Fed Median (IQR) = 503 (266.2, 660).

** Nonparametric data, median (IQR) = 321 (224–1,709). For comparison AUC Fed, Median IQR = 2816 (1763, 3160).

## Discussion

This study demonstrates that oral administration of an FDA-approved pure CBD product in fasted cats at a dose of 5 mg/kg resulted in detectable CBD concentrations in plasma. A statistically significant difference was noted between the fed and fasted AUC, a parameter that represents the extent of absorption, suggesting that this CBD medication should be administered shortly after a meal. Interestingly, two cats displayed higher concentrations in the fasted compared to the fed state. Although care was taken to assure that the complete dose was ingested in all cats, these two cats did not receive the complete dose when they were in the fed portion of the study.

Both the mean C_max_ and AUC of CBD in plasma in this study were higher when compared to other studies with similar dosage administration (26–28). A summary of previous pharmacokinetic parameters from other studies is presented in Table 2. Several reasons could be used to explain these higher concentrations. First and foremost, CBD was administered after feeding in this study, which has been shown to significantly increase the absorption of CBD in people (16). However, concentrations reported for fasted cats in our study are also higher. Differences in the products themselves may contribute to differences in exposure after oral administration due to differences in bioequivalence. Cannabinoids are very lipophilic and must be prepared in oil bases, which may alter bioavailability (29). Differences in the oil vehicles and excipients may have contributed to differences in absorption. Epidiolex^®^ is a medical-grade cannabidiol product, with sesame seed oil as the vehicle, but also contains dehydrated alcohol, strawberry flavor, and sucralose (16). Product quality may also be contributing to differences. Labeling inaccuracies have been documented to occur in both veterinary and human CBD supplements by measuring product CBD concentration and comparing results to those listed on the product label (18, 30).

**Table 2:** Summary of the current and previously reported pharmacokinetic parameters for single dose of CBD administered to cats.

**Parameter**	**Present study Mean ±SD**	**Present study Mean ±SD Fed**	**Wang et al. (28) 24 h Mean ±SD**	**Deabold et al. (27) Mean ±SEM**	**Rozental et al**. **(****29****)** **Mean (Range)**					
Number of cats in study group	*n =* 8	*n =* 8	*n =* 8	*n =* 5	*n =* 4	*n =* 4	*n =* 4	*n =* 4	*n =* 4	*n =* 4
Dose mg/kg	5	5	1.37 ± 0.15	2	2.5	5	10	20	40	80
Cmax (ng/mL)	269 ± 334 ^*^65 (16.9–865.1)	465.3 ± 220	282 ± 149.4	43 ± 9	17.8 (3.2–45.3)	61.1 (19.9–148.5)	132.6 (43.2–258.4)	281.0 (14.5–467.4)	251.7 (47.4–467.0)	963.9 (744.6–1126.8)
Tmax (hours)	2.6 ± 1.6	4.7 ± 2.1	2 ± 0	2.0 ± 0.6	^*^2.0 (2.0–4.0)	^*^2.0 (2.0–4.0)	^*^2.0 (2.0–24.0)	^*^2.0 (2.0–4.0)	^*^2.0 (2.0)	^*^3.0 (2.0–6.0)
Disapperance Half Life (hours)	4.1 ± 4.4	5.9 ± 2	2.1 ± 1.1	1.5 ± 0.1	13.2 (12.8–13.5)	8.2 (6.0–11.0)	7.5 (6.2–8.5)	9.0 (5.7–14.9)	9.6 (6.3–17.7)	6.7 (6.6–6.7)
AUC (ng^*^h/mL)	921 ± 1,003 ^*^321 (161.9–2,858.5)	2,650 ± 1,188	908.5± 528.1	164 ± 29	83.5 (8.1–165.9)	437.1 (180.0–1,139.2)	1,000.4 (460.8–1,714.1)	1,481.0 (92.9–2,372.9)	1,945.8 (313.1–4,150.1)	8,738.1 (4,269.6–10,690.0)
MRT (h)	12.1 ± 7.5	10.2 ± 2.4	3.8 ± 1.0	3.5 ± 1.4	4.5 (2.2–8.5)	7.0 (5.9–8.1)	9.0 (7.0–13.9)	6.3 (7.2–8.1)	7.5 (8.1–15.1)	8.4 (7.6–9.4)
VdF (L/kg)	668.3 ± 1,130	76.5 ± 66.8	Not reported	Not Reported	239.7 (239.6–239.8)	196.9 (50.2–344.7)	110.1 (65.1–179.1)	886.6 (73.1–3246.3)	702.0 (89.0–2152.5)	68.3 (65.8–70.9)
CL/F (L/h/kg)	16 ± 10.1	2.7 ± 2.3	Not reported	Not Reported	12.6 (12.3–13.0)	17.4 (3.7–23.1)	11.1 (5.3–20.2)	45.2 (8.5–84.6)	37.3 (7.7–71.8)	7.13 (6.8–7.5)

Among the questions that need to be answered regarding the use of CBD to control seizures is the effective concentration of CBD. The answer to this question is complicated by the lack of evidence regarding exactly how CBD imparts an antiseizure effect. CBD in the form of Epidiolex^®^ has demonstrated efficacy for the treatment of selected causes of refractory epilepsy in children, which is demonstrated by its approval as a drug for that indication (16). Although a reference interval for CBD that could help guide therapeutic decision-making has not been established, for any species, recent data suggest that epileptic seizure control was improved with each 100 ng/mL concentration of CBD increase (31). In a veterinary trial of CBD for the add-on treatment of epilepsy, two dogs in the CBD group demonstrated at least a 50% reduction in seizure frequency and had plasma drug concentrations >400 ng/mL (32). Additionally, higher CBD doses administered to rats resulted in improved protection compared to the administration at lower doses in an electroshock model for seizures (9). Although the clinical importance of achieving higher concentrations of CBD in cats is unknown at this time, C_max_ concentrations, demonstrated in this study, may be sufficiently high enough to exert an effect.

When designing a dosing regimen, it is important to consider both the pharmacokinetic and pharmacodynamic effects of a drug. Given that the mechanism of action for seizure control of CBD medications is not entirely understood, relying on pharmacokinetic data, in particular a terminal half-life of around 5 h, suggests that a dosing interval of at least 8–12 h may be necessary. However, even at an 8-h dosing interval, blood concentrations may fluctuate by over 50%. One of the limitations of this study is that disposition was studied after only a single dose. Whether or not changes in disposition over time might contribute to the need for dose adjustment is not known.

Using an FDA-approved product rather than products that do not require a prescription (such as dietary supplements) offers several advantages. Foremost, perhaps, is any question regarding the quality of the product. The FDA requires that drug manufacturers adhere to Current Good Manufacturing Practice (CGMP) regulation.^1^ These guidelines ensure that particular specifications are used throughout the manufacturing process, that the product is safe for use, and that label claims are accurate. Second, the concentration of CBD in the product studied here is 100 mg/mL. This concentration allows for smaller, and presumably more convenient, dosing volumes. Lastly, yet equally important, FDA-approved medications go through a rigorous process to ensure safety. One downside to the use of an FDA-approved product can be the high cost, especially when considering the per-unit price. However, when comparing to multiple veterinary products, the cost per 10 mg of CBD was well within the range of prices associated with products being marketed for animals.

In conclusion, this study demonstrates that a single dose of 5 mg/kg of a CBD medication, given as the FDA human-approved drug and administered with food, safely achieved, in feline plasma, CBD concentrations that may achieve the desired therapeutic effect. Although it is unknown whether this concentration will have an effect is unknown, it may serve as a good starting point based on the available literature. Future studies assessing multiple-dose pharmacokinetics and therapeutic trials should be performed to help establish a dosing regimen.

## Data availability statement

The raw data supporting the conclusions of this article will be made available by the authors, without undue reservation.

## Ethics statement

The animal study was reviewed and approved by Auburn University Animal Care and Use Committee.

## Author contributions

TJ: experimental design, sample processing, statistical analysis, and manuscript preparation. DB: experimental design, pharmacokinetic analysis, and manuscript preparation. CC-E: analytical chemist, method developing and validation, CBD extraction from the sample, mass specification analysis, and interpretation. DM: IACUC submission, animals used in the study, and manuscript preparation. All authors contributed to the article and approved the submitted version.

## Footnotes

^1^Available online at: https://www.fda.gov/drugs/pharmaceutical-quality-resources/current-good-manufacturing-practice-cgmp-regulations.
