# Supplementary material for: Disposition of a single oral dose of a cannabidiol medication in healthy cats
Source: Front Vet Sci. 2023 May 26;10:1181517. doi: 10.3389/fvets.2023.1181517 (PMC10251743; doi:10.3389/fvets.2023.1181517)
Supplement: Supplementary file 1 [file Table_1.DOCX]

Supplementary Material

| **Parameter** | **Mean** | **Reference Interval** |
| --- | --- | --- |
| Total Protein | 7.662 | 6.20 - 7.70 |
| Albumin | 3.287 | 2.80 - 4.20 |
| Globulin | 4.36 | 2.40 - 4.40 |
| Albumin/Globulin | 0.803 | 0.50 - 1.30 |
| ALP | 28.1 | 24 - 75 |
| ALT | 61 | 26 - 77 |
| AST | 44.3 | 12 - 45 |
| Tbili | 0.026 | 0.10 - 0.20 |
| CK | 356.8 | 100 - 250 |
| BUN | 22.29 | 5.0 - 30.0 |
| Cre | 0.96 | 0.0 - 2.0 |
| Calcium | 9.26 | 9.5 - 11.6 |
| Phosphorous | 4.42 | 4.3 - 5.9 |
| Glucose | 114.8 | 58 - 116 |
| Chol | 132.5 | 88 - 294 |
| HCO3 | 16.83 | 12.0 - 31.0 |
| Na | 148.3 | 145 - 158 |
| K | 3.38 | 3.3 - 5.7 |
| Cl | 116.9 | 114 - 120 |
| AG | 17.95 | 17.0 - 29.0 |
| Osmol | 296.5 | 308 - 325 |
| S. Iron | 83.4 | 48 - 175 |
| Lipemia Index | 20.6 | 0 - 8 |
| Hemolysis Index | 291.6 | 0 - 35 |
| Icterus Index | 0 | 0 - 0 |
| UIBC | 203.4444444 | 123 - 430 |
| TIBC | 280.8888889 | 243 - 553 |

Serum biochemistry from cats included in the study
